# Supplementary material for: Do exercise-associated genes explain phenotypic variance in the three components of fitness? a systematic review & meta-analysis
Source: PLoS One. 2021 Oct 14;16(10):e0249501. doi: 10.1371/journal.pone.0249501 (PMC8516263; doi:10.1371/journal.pone.0249501)
Supplement: S1 Fig — The results of both reviewers using the quality assessment tool is mapped as the difference in scores against the average score (Bias). The 95% LoA are also calculated and represented as the upper and lower 1.96 dashed lines. The confidence intervals for the 95% LoA were calculated using Bland Altman’s approximate method. (PDF) [file pone.0249501.s001.pdf]

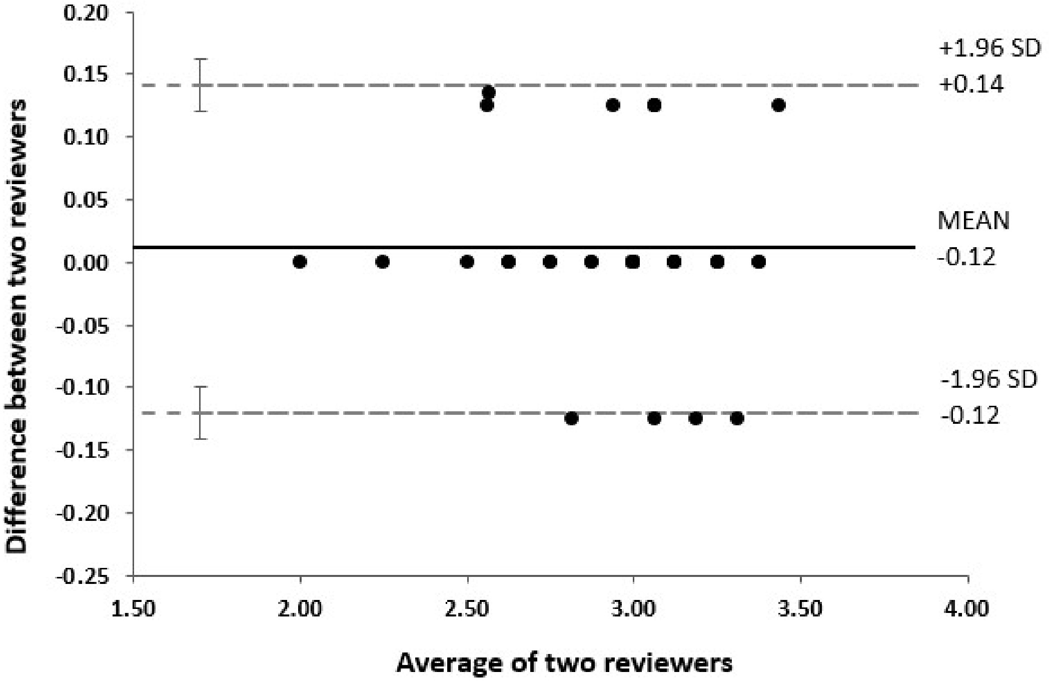

S1 Fig. Bland Altman plot. The results of both reviewers using the quality assessment tool is mapped as the difference in scores against the average score (Bias). The 95% LoA are also calculated and represented as the upper and lower 1.96 dashed lines. The confidence intervals for the 95% LoA were calculated using Bland Altman's approximate method.
