# Supplementary material for: Do exercise-associated genes explain phenotypic variance in the three components of fitness? a systematic review & meta-analysis
Source: PLoS One. 2021 Oct 14;16(10):e0249501. doi: 10.1371/journal.pone.0249501 (PMC8516263; doi:10.1371/journal.pone.0249501)
Supplement: S1 Table — Search terms implemented for all the databases and the number of results shown by hits. (PDF) [file pone.0249501.s002.pdf]

S1 Table. Search terms and results. Search terms implemented for all the databases and the number of results shown by hits.

| Data Base | Hits | Search Terms |
|---|---|---|
| **Scopus** | 3,260 | TITLE-ABS-KEY ( "Gene" OR "Genes" OR genetic* OR geno* OR genotype* OR mutation* OR phenotype* OR dna OR rna OR allele* OR chromosome* OR haplotype* OR haplogroup* OR snp* OR polymorphism* OR heterozygous OR homozygous OR "Deoxyribonucleic acid" OR "Ribonucleic acid" OR nucleotide OR "epigenetics" OR "methylation" OR "demethylation" OR "acetylation" OR "deacetylation" ) AND ( "Exercise intervention" OR "Exercise training" OR "Exercise program*" OR "Exercise session" OR "Exercise regime*" OR "Training program*" OR "Training intervention" OR "Training session" OR "Training regime*" OR "Physical activity program*" OR "Physical activity intervention" OR "Physical activity session" OR "Fitness intervention" OR "Fitness program*" OR "Fitness training" OR "Sport* intervention" OR "Sport* training" OR "Training weeks" OR "Intervention weeks" OR "Exercise weeks" ) AND ( "VO2*" OR "Cardiovascular endurance" OR "Aerobic fitness" OR "Maximal Aerobic Power" OR "Muscle strength" OR "1RM" OR "1 repetition maximum" OR "Anaerobic Power" OR "Peak power output" OR "PPO" ) |
| **Web of Science** | 318 | ((TS=("Gene") OR TS=("Genes") OR TS=(Genotype*) OR TS=(Geno*) OR TS=(Mutation*) OR TS=(Phenotype*) OR TS=(DNA) OR TS=(RNA) OR TS=(Allele*) OR TS=(Chromosome*) OR TS=(Haplotype*) OR TS=(Haplogroup*) OR TS=(SNP*) OR TS=(Polymorphism*) OR TS=(Heterozygous) OR TS=(Homozygous) OR TS=(Deoxyribonucleic acid) OR TS=(Ribonucleic acid) OR TS=(Nucleotide) OR TS=(Epigenetics) OR TS=(Methylation) OR TS=(Demethylation) OR TS=(Acetylation) OR TS=(Deacetylation)) AND (TS=("Exercise intervention") OR TS=("Exercise training") OR TS=("Exercise program*") OR TS=("Exercise session") OR TS=("Exercise regime*") OR TS=("Training program*") OR TS=("Training intervention") OR TS=("Training session") OR TS=("Training regime*") OR TS=("Physical activity program*") OR TS=("Physical activity intervention") OR TS=("Fitness intervention") OR TS=("Fitness program*") OR TS=("Fitness training") OR TS=("Sport* intervention") OR TS=("Sport* training") OR TS=("Training weeks") OR TS=("Intervention weeks") OR TS=("Exercise weeks")) AND (TS=("VO2*") OR TS=("Cardiovascular endurance") OR TS=("Aerobic fitness") OR TS=("Maximal Aerobic Power") OR TS=("Muscle strength") OR TS=("1RM") OR TS=("1 repartition maximum") OR TS=("Anaerobic Power") OR TS=("Peak power output") OR TS=("PPO"))) |

| | | |
|---|---|---|
| **Pubmed** | 180 | ((((“Gene”[Title/Abstract] OR “Genes”[Title/Abstract] OR Genetic*[Title/Abstract] OR Geno*[Title/Abstract] OR Genotype*[Title/Abstract] OR Mutation*[Title/Abstract] OR Phenotype*[Title/Abstract] OR DNA[Title/Abstract] OR RNA[Title/Abstract] OR Allele*[Title/Abstract] OR Chromosome*[Title/Abstract] OR Haplotype*[Title/Abstract] OR Haplogroup*[Title/Abstract] OR SNP*[Title/Abstract] OR Polymorphism*[Title/Abstract] OR Heterozygous[Title/Abstract] OR Homozygous[Title/Abstract] OR “Deoxyribonucleic acid”[Title/Abstract] OR “Ribonucleic acid” [Title/Abstract] OR Nucleotide[Title/Abstract] OR "Epigenetics"[Title/Abstract] OR "Methylation"[Title/Abstract] OR "Demethylation"[Title/Abstract] OR "Acetylation"[Title/Abstract] OR "Deacetylation"[Title/Abstract])) AND (“Exercise intervention”[Title/Abstract] OR “Exercise training”[Title/Abstract] OR “Exercise program*”[Title/Abstract] OR “Exercise session”[Title/Abstract] OR “Exercise regime*”[Title/Abstract] OR “Training program*”[Title/Abstract] OR “Training intervention”[Title/Abstract] OR “Training session”[Title/Abstract] OR “Training regime*”[Title/Abstract] OR “Physical activity program*”[Title/Abstract] OR “Physical activity intervention”[Title/Abstract] OR “Physical activity session”[Title/Abstract] OR “Fitness intervention”[Title/Abstract] OR “Fitness program*”[Title/Abstract] OR “Fitness training”[Title/Abstract] OR “Sport* intervention”[Title/Abstract] OR “Sport* training”[Title/Abstract] OR “Training weeks”[Title/Abstract] OR “Intervention weeks”[Title/Abstract])) AND ("VO2*"[Title/Abstract] OR “Cardiovascular endurance”[Title/Abstract] OR “Aerobic fitness”[Title/Abstract] OR “Maximal Aerobic Power”[Title/Abstract] OR “Muscle strength”[Title/Abstract] OR “1RM”[Title/Abstract] OR “Anaerobic Power”[Title/Abstract] OR “Peak power output”[Title/Abstract] OR “PPO”)) |
| **SPORTDiscus** | 202 | ( “Gene” OR “Genes” OR Genetic* OR Geno* OR Genotype* OR Mutation* OR Phenotype* OR DNA OR RNA OR Allele* OR Chromosome* OR Haplotype* OR Haplogroup* OR SNP* OR Polymorphism* OR Heterozygous OR Homozygous OR “Deoxyribonucleic acid” OR “Ribonucleic acid” OR Nucleotide OR "Epigenetics" OR "Methylation" OR "Demethylation" OR "Acetylation" OR "Deacetylation" ) AND ( “Exercise intervention” OR “Exercise training” OR “Exercise program*” OR “Exercise session” OR “Exercise regime*” OR “Training program*” OR “Training intervention” OR “Training session” OR “Training regime*” OR “Physical activity program*” OR “Physical activity intervention” OR “Physical activity session” OR “Fitness intervention” OR “Fitness program*” OR “Fitness training” OR “Sport* intervention” OR “Sport* training” OR “Training weeks” OR “Intervention weeks” OR “Exercise weeks” ) AND ( "VO2*" OR “Cardiovascular endurance” OR “Aerobic fitness” OR “Maximal Aerobic Power” OR “Muscle strength” OR “1RM” OR “Anaerobic Power” OR “Peak power output” OR “PPO” ) |

- TITLE-ABS = title and abstract; TS = Topic; * = truncation; “ “ = phrase or joint word; ‘OR’ & ‘AND’ = Boolean operators/logic; HITS = number of results.
