# Supplementary material for: Do exercise-associated genes explain phenotypic variance in the three components of fitness? a systematic review & meta-analysis
Source: PLoS One. 2021 Oct 14;16(10):e0249501. doi: 10.1371/journal.pone.0249501 (PMC8516263; doi:10.1371/journal.pone.0249501)
Supplement: S2 Table — The average score between reviewers was taken for the final inclusion. Power threshold was based at 0.8 (80%). (PDF) [file pone.0249501.s003.pdf]

S2 Table. COSMIN assessment tool and Post-Hoc power. The average score between reviewers was taken for the final inclusion. Power threshold was based at 0.8 (80%).

Study	Reviewer 1 COMIN score	Reviewer 2 COMIN score	Mean scores of reviewers	Included in review (Yes/No)	Statistical Power (ß) (%)
Ahtiainen et al., 2011	3.38	3.25	3.31	Yes	**46.3**
**Bae et al., 2007**	**2.50**	**2.64**	**2.57**	**No**	81.3
Böhm et al., 2016	3.25	3.25	3.25	Yes	**25.4**
Bouchard et al., 2010	3.00	3.00	3.00	Yes	89.2
Butcher et al., 2008	3.13	3.13	3.13	Yes	13.6
**Caldow et al., 2015**	**2.63**	**2.63**	**2.63**	**No**	98.1
**Camera et al., 2010**	**2.50**	**2.63**	**2.56**	**No**	**NR**
**Christiansen et al., 2010**	**2.75**	**2.75**	**2.75**	**No**	**53.6**
**Cięszczyk et al., 2016**	**2.75**	**2.75**	**2.75**	**No**	96
Clarkson et al., 2005	3.00	3.00	3.00	Yes	100
Colakoglu et al., 2005	3.25	3.13	3.19	Yes	100
Davidsen et al., 2010	3.13	3.00	3.06	Yes	**78.8**
**Denham et al., 2018**	**2.50**	**2.50**	**2.50**	**No**	**54.5**
Dohlmann et al., 2018	3.25	3.25	3.25	Yes	84.3
Egan et al., 2013	3.00	3.00	3.00	Yes	**77.4**
Erskine et al., 2012	3.13	3.13	3.13	Yes	96.1
Gentil et al., 2011	3.25	3.25	3.25	Yes	85
Gentil et al., 2012	3.38	3.50	3.44	Yes	98.3
**Hamel et al., 1986**	**2.88**	**2.88**	**2.88**	**No**	**54.7**
**Harmon et al., 2010**	**2.88**	**2.88**	**2.88**	**No**	100
He et al., 2010	3.00	3.00	3.00	Yes	**46.8**
Kazior et al., 2016	3.00	3.00	3.00	Yes	99.9
Konopka et al., 2013	3.00	3.13	3.06	Yes	84.1
Lamas et al., 2010	3.00	3.00	3.00	Yes	99.7
Little et al., 2010	3.00	3.00	3.00	Yes	84.2
Lundberg et al., 2014	3.00	3.13	3.06	Yes	**75.4**
McPhee et al., 2011a	3.13	3.13	3.13	Yes	89.3
McPhee et al., 2011b	3.00	3.13	3.06	Yes	100
**Murakami et al., 2002**	**2.88**	**2.75**	**2.81**	**No**	99.9
**Nader et al., 2014**	**2.63**	**2.63**	**2.63**	**No**	100
**Parcell et al., 2005**	**2.63**	**2.63**	**2.63**	**No**	97.6
Parra et al., 2000	3.00	3.00	3.00	Yes	100
Rankinen et al., 2000a	3.38	3.38	3.38	Yes	100
Rankinen et al., 2000b	3.25	3.25	3.25	Yes	100
Rico-Sanz et al., 2004	3.00	3.00	3.00	Yes	100
**Schrauwen et al., 2002**	**2.00**	**2.00**	**2.00**	**No**	**19.4**
Silva et al., 2015	3.00	3.13	3.06	Yes	90.7
Thomis et al., 2004	3.38	3.38	3.38	Yes	97.1

Thompson et al., 2004	3.13	3.13	3.13	Yes	97.6
**Walker et al., 2004**	**2.88**	**3.00**	**2.94**	**No**	99.9
Wilkinson et al., 2008	3.13	3.13	3.13	Yes	100
Yu et al., 2014	3.00	3.00	3.00	Yes	86.8
**Zarebska et al.,2014**	**2.25**	**2.25**	**2.25**	**No**	81.1

- Bold highlight = did not meet the threshold scores; NR = not reported (unable to calculate power due to lack of data presented).
