# Supplementary material for: Do exercise-associated genes explain phenotypic variance in the three components of fitness? a systematic review & meta-analysis
Source: PLoS One. 2021 Oct 14;16(10):e0249501. doi: 10.1371/journal.pone.0249501 (PMC8516263; doi:10.1371/journal.pone.0249501)
Supplement: S1 File — (PDF) [file pone.0249501.s004.pdf]

Meta-analysis on Genetic Association Studies Checklist | PLOS ONE

	Item	Section name and paragraph number within manuscript
	**Introduction**	
1	Provide a detailed justification for the polymorphism studied; if a single polymorphism was analyzed, give details as to why others were not included in the meta-analysis.	Identified in the Results section, line number 168 and shown in figures 2, 3 and 4.<br>One aim of the MA was to idenfiy the many genes and polymorphisms associated to the three componenets of fitness, therefore, details of them were not in the introduction.<br>Why other genes were not included is reffered to in the limitations section line 410.
2	Provide a detailed justification for the population(s) and clinical condition studied.	Justification of the group is in the introduction lines 62, 86 and 93. Expanded and identified in the Methods section lines 115-120.
	**Methods**	
3	Provide full details of the search strategy employed; outline the full electronic search strategy –specific combination of keywords and any limits applied- for at least one database. Specify whether synonyms of polymorphisms/genes (e.g. SNP number) were searched.	The exact searches, keywords and terms used is provided in the supplimentary document 'supporting information' table S1. This included boolean operators, synonyms and other search technques.<br>This is breifly mentioned within the manuscript in the methods section first paragraph.
4	Report full details on the inclusion and exclusion criteria applied for selecting studies. *Please list the excluded articles and the reasons for exclusion of each article in a supplementary file.*	Eligibility criteria section includeds PICO stratrergy which was used as trhe criteria for inclusion and exclusion (lines 115-129).

5	Provide details on how the quality of the studies included in the analyses was assessed.	COSMIN check list (lines 130-142) results are in 'supporting information' document table S2.
6	Describe steps taken to contact study authors to identify additional studies and to request missing data.	Methods section lines 108-110.
7	Describe how environmental effects were adjusted for, if this adjustment was not conducted, outline the reasons for this.	NA, however, the study effects were adujest for and explained in lines 398-406.
8	Describe the methods of handling heterogeneity/between-study variance.	Heterogeneity was assessed and stated in lines 158-161 in statisitcal analysis section. This was controlled with subgroup analysis lines 161-164 and explained in discussion section lines 398-406.
9	Describe how the Hardy-Weinberg equilibrium and linkage disequilibrium were assessed.	NA
10	Describe and justify the choice of model for the analyses (per-allele vs per-genotype vs genetic model-free, random effects vs fixed effects).	Statsitical methods section briefly explains how the data was handled. Dicussion section lines 312-314 and 347-348 explain limitions of studies forcing a per genotype vs genotype approach.
11	Describe whether a sensitivity analysis has been completed.	The study search and flow diagram was repeated as explained in figure 1 caption. COSMINS was assessed twice for sensitive outcomes S2.
12	Describe whether an assessment of the effects of population stratification has been conducted.	conducted in Stasticial methods section (line 156).
13	Describe whether study-specific results have been assessed and if so the reasons for this (e.g. forest plot).	Described and explained in the Statsirtical methods section.
	**Results**	
14	Include flow diagram for the studies included in the meta-analysis as the first figure for the manuscript	Figure one is uploaded seperatley at Fig 1 (tiff file) this is addressed in results section line 168.

15	Report details on allele/genotype prevalence.	Results section, figures 2, 3, 4 and tables 1, 2, 3.
16	Report the effect size estimates and p values for each analysis.	Results section, Figures 2, 3 and 4 and lines 188-193, 226-230 and 248-251.
	**Discussion**	
17	Discuss the limitations of the meta-analysis, including genotyping errors/bias and publication bias.	Discussion section lines 412-431.
18	If the meta-analysis identifies an association within a subgroup of the population studied but not another, discuss the implications of these results, and if applicable the possibility of subgroup-specific publication bias.	NA
19	Discuss the suitability of the sample size employed to the research question and the power of the study.	NA
