# Supplementary material for: Do exercise-associated genes explain phenotypic variance in the three components of fitness? a systematic review & meta-analysis
Source: PLoS One. 2021 Oct 14;16(10):e0249501. doi: 10.1371/journal.pone.0249501 (PMC8516263; doi:10.1371/journal.pone.0249501)
Supplement: Attachment — Submitted filename: Response to Reviewers 1.docx [file pone.0249501.s006.docx]

Rebuttal letter ‘Response to Reviewers' comments

| Academic editor and reviewer(s) points | Response to comments |
| --- | --- |
| Reviewer 1: | Authors: |
| Abstract is well written yet I would like to see you denote the gender breakdown of participants as well as their mean age. | This information has been added to the abstract. |
| Also, since this is in untrained participants, is this really that important considering that these individuals are not using these outcomes on a day-to-day basis? Does the merit of this then solely relate to training studies performed in this sample trying to better understand how these factors changes in response to various training regimens? | The introduction outlines how important these health-related components of fitness are for all people, particularly in the untrained. Here even small improvements can help untrained people complete common everyday tasks more easily.  What this study aims to do is to see how we can best maximise improvements in these health-related components and also if genetics play a significant role in determining improvements in these components of fitness. The results clearly show that the differences in improvements, between participants, are gene related: up to 72% in some aspects. |
| Introduction: Lines 52-56 should also denote that VO2max is a correlate or morbidity and mortality according to Blair et al. (1996), etc. | This has been added and reference numbers revised, accordingly. |
| Line 57: isn’t strength the maximal force generated by muscle? | This is the general definition of strength that is needed for everyday tasks, rather than maximal strength. We have edited the wording to reflect generated instead of “perform”. |
| Line 73: but my recollection of these data which is a little foggy is that the relationships (R2 value) between these genes and measures such as VO2max are not that strong e.g. < 20 %, so clearly other attributes have a stronger association with this measure than a gene or two. | I have edited this slightly and removed these two specific genes and tried to make this more general.  Your statement is partly correct. Things such as training loads have significantly higher correlations with improvements in VO2max. However, genetics still exhibits significant associations with the improvements in the phenotype and this is well-supported in the literature. E.g. Heritage study found up to 50% of the differences in improvements in VO2max were explained by the genes, which individually could be up to 15% and highly significant, even more so in strength and power phenotypes figures. |
| Line 93: is a hypothesis merited here? | We have considered this and do not believe so. We think for this review there is no merit of a hypothesis. |
| Methods; line 117: is non-obese also an inclusion criterion? Please describe. And were studies only included if they did not have any nutritional intervention such as protein intake, etc. to optimize changes in strength? | Edited. We have stated that the participant population are healthy untrained males and females with no recorded medical conditions.  I have added “(f) no dietary manipulation” in the inclusion criteria. Studies were only included if there was no dietary intervention. |
| Line 120: is this treadmill derived VO2max or that on the cycle ergometer? And I assume that this is leg press derived measures of 1RM and Wingate test derived indices of Anaerobic power? For example, load determines PPO from the Wingate test so if a study used a relatively light training load, then resultant PPO is lower. Is this a limitation of your criteria? | Yes, this is cycle ergometer derived VO2max, 1RM is leg-press and PPO is Wingate. We have addressed the limitations of measuring Power via Wingate test at the end of the discussion section. However, training load is only relative to the same type of exercise, and this is a problem for strength and power. If you use reps and sets rather than time, like endurance training load, it’s always different and non-comparable as they input variables are different. Therefore, you can only really compare training loads if the input variables are the same, i.e intensity, duration, frequency between studies are measured in the same units. The most important point to note is that the PPO measure is always somewhat arbitrary and as long as the power protocol used is similar, power can be compared across power studies, which this study does. |
| Study selection: were studies included and excluded using some software, or did 2 or 3 of the authors meet and talk about whether a study was included or not? Please explain this in the text and how any disagreements were handled; thank you. | The criteria for study selection and inclusion were met if the papers met the COSMIN scores. This is highlighted in the Quality and Risk of Bias Assessment section, however, we have edited to improve clarity. |
| Results, line 178: can you please add to your text here the range of training duration of these studies eliciting this near 11 % increase in CRF; thank you. And please do the same for the change in strength and AN power. | We have added the average training durations, intensities, and frequencies for CRF now. With strength we have now included number of reps, intensities and frequencies and AN Power with number of exercise bouts and duration. |
| Table 1: can you please explain here in the legend what ‘mean rank’ refers to, as you did with the other outcome = subgroup? | This has now been added to all relevant tables. |
| Discussion, line 267: Please finish this section of text here with a brief argument or rationale as to why this review and its findings matter to the broader field of Sports Science. | Text revised accordingly. Thanks for this helpful comment. |
| Line 272: I do not do this genetic work, so this may be a dumb question, but what does it mean when a single gene is related to training responsiveness for each of these 3 outcomes which are physiologically limited by different factors i.e. O2 delivery for CRF, muscle mass, FT fiber %, and neuromuscular function for 1RM and PPO? Does gene expression change based on the training volume or duration performed in these studies? If so, how does this affect your resultant data? | This is a very good question.  For the first part, the literature has shown that certain genes can have multiple responses i.e the alternative I/D alleles, of the ACE gene, can influence both aerobic and anaerobic performance. Thus, DD carriers tend to be advantaged in strength, whilst II allele carriers generally show better improvements in endurance performance. In terms of untrained participants however, it has recently been shown that these mixed results make sense because improvements in strength would still indirectly benefit aerobic performance because of the increase in muscle mass allowing more O2extraction (a-vO2 diff), further increases, in say, leg strength would help cycle ergometer performance i.e time to exhaustion (true VO2max) and PPO.  The main problem with many exercise-genetic relationship studies, which is identified in our study, is that they do not identify specific alleles, which can often confound findings. This is something we have tried to address, where possible, in this study.  Secondly, there is good evidence that exercise can affect gene transcription. Epigenetic factors are also responsive to exercise, but this paper was not aimed at identifying genes that may be transcriptionally regulated by exercise, rather identifying those genes associated with training improvement, regardless of their modus operandi. Therefore, in the presence of training the genotype subgrouping is only for subgrouping and the data does not change regardless of exercise as there is no gene information reported other than the gene grouping and the scores of the primary variables of interest. |
| Line 322: ‘equivocal results’ for what outcome or factor? | Edited and added both for VO2max and 1RM. |
| Line 353: I believe this link needs to be replaced with a reference citation. | We have edited this accordingly and also edited the reference numbers for those that follow so that the order of the references matches the reference list and in-text order. |
| Reviewer 2: |  |
| While the authors do address the role of each candidate gene, further discussion into the context of their role regarding previous publications should be made (e.g., gene knockout/ in studies).  Moreover, as genome wide analysis in response to training interventions are becoming more frequent, the authors should discuss their findings in context of these. | This is an important point, and one we should have made clearer in the text. This analysis was looking at Human studies only. Gene knockout is not something done in Humans, for very sound ethical reasons, only in animal models, such as mice, rats etc. In terms of human studies, we feel that the reference to the gene’s role and the variants have been established in the discussion using the current references. Therefore, this is not something we can reasonably address within this study.  GWAS has proved very important within genetic studies in identifying novel disease associations in the many variants of particular genes, both in coding and non-coding (e.g. Introns) sequences. However, a key limitation of GWAS is that most of the variants do not have any links to a biological response, largely due to code degeneracy and that GWAS is typically only helpful when identifying new variants (Al-Khelaifi et al 2020). Moreover, as already noted, the influence of individual genes and their alleles is complex as they can interact to produce the final phenotypic outcome and should not be compared in isolation (Tam et al., 2019).  To try and address this point we have added text at the start of the discussion, and we hope it strengthens the manuscript in this respect, as well as meeting your question. |
| Line 364 – This line is potentially misleading. While testosterone plays a role, AKT/mTOR are the key regulators of muscle mass/strength. | We have edited this part accordingly to reflect this comment. |
| Lines 351-357 – The reason for these lines are unclear, while mTOR polymorphisms may have a role in response to endurance vs strength bases training, these were not assessed in the studies analysed. | We have modified the text to address your comment. The point is that the mTOR polymorphisms have multiple effects, requiring an allele specific role analysis which was not in the papers included in this study. |
| Lines 412- 414 – The first limitation is unclear. | We agree, with this comment and edited the text, accordingly, noting that the main limitation, which is the lack of allele specific analysis. |
| Line 436-437 – Could the authors clarify this sentence; it is currently speculative to tailor training interventions based upon an individual’s genotype. | Whilst this review supports the notion that genotype specific training programmes might be more advantageous than generic training, indeed this is currently what commercial genetic companies try to do, such as Muhdo Health Ltd. We have edited this to say “in theory”. |
| Table S2 – Could the authors provide numbered references against each of the studies. | The tables do not include any studies to reference, as these are the subgroup analysed via genotype.  We assume this is for all the figures? So have now changed all the figures to have the numbered references. |
